# Hybrid Tele-Rehabilitation in the Management of Pediatric Chronic Suppurative Lung Diseases: Study Protocol for a Randomized Controlled Trial

**DOI:** 10.3390/healthcare14091250

**Published:** 2026-05-06

**Authors:** Aspasia Mavronasou, Panagiotis Dalamarinis, Arietta Spinou, Dafni Moriki, Konstantinos Douros, Eleni A. Kortianou

**Affiliations:** 1 Clinical Exercise Physiology & Rehabilitation Research Laboratory, Physiotherapy Department, School of Health Sciences, University of Thessaly, 35132 Lamia, Greece; asmavronasou@uth.gr; 2 “Paediatric Respiratory Medicine” MSc Program, Medical School, National and Kapodistrian University of Athens, 10679 Athens, Greece; pdalamarinis@med.uoa.gr; 3 School of Life Course & Population Sciences, Faculty of Life Sciences & Medicine, King’s College London, London WC2R 2LS, UK; arietta.spinou@kcl.ac.uk; 4 King’s Centre for Lung Health, King’s College London, London WC2R 2LS, UK; 5 Pediatric Allergy and Pulmonology Unit, 3rd Department of Pediatrics, National and Kapodistrian University of Athens, 10679 Athens, Greece; dmoriki@med.uoa.gr (D.M.); kdouros@med.uoa.gr (K.D.)

**Keywords:** chronic suppurative lung diseases, internet-based intervention, pediatrics, physiotherapy, telerehabilitation

## Abstract

**Background:** The pediatric population with chronic suppurative lung diseases (CSLDs) presents with a clinical profile of persistent productive cough and impaired airway clearance, which leads to reduced exercise capacity and physical activity (PA). The main objective of this research is to evaluate the effect of a 12-week intervention that combines airway clearance techniques (ACTs) and exercise training delivered through synchronous (videoconferencing) and asynchronous implementation at home, supported by an informative, specially designed website and supervised by a physiotherapist on exercise capacity. The secondary objective is to assess adherence to the ACT performance. The hypothesis of the study is that this hybrid tele-rehabilitation program will improve functional and exercise capacity, as well as compliance with ACTs, compared to the usual care. **Methods**: Thirty-two children with CSLDs (other than cystic fibrosis) will be randomly assigned to either the intervention group (home-based, synchronous, and asynchronous ACTs and exercise training) or the control group (usual care). An assessor blind to group allocation will measure the modified shuttle walk test, 6-minute walking test, Chester step test, respiratory muscle strength, handgrip, pulmonary function, PA, sedentary behavior, and quality of life at baseline, at the end of the intervention (3 months), and 6 months after completion. **Discussion**: This study introduces a hybrid (synchronous and asynchronous) tele-rehabilitation program, aiming to improve physical capacity and adherence to physiotherapy management for pediatric CSLD populations.

## 1. Introduction

Chronic suppurative lung diseases (CSLDs) are increasingly recognized as significant contributors to pediatric respiratory morbidity. Within the CSLD’s spectrum, conditions such as protracted bacterial bronchitis (PBB), primary ciliary dyskinesia (PCD), and non-cystic fibrosis bronchiectasis (NCFB) are clinically unified by the hallmark symptom of chronic wet cough [1]. The prevalence of these disorders is highly contingent on diagnostic access and socioeconomic factors. In European populations, the estimated annual incidence ranges from 0.2 to 2.3 per 100,000 children, whereas non-European cohorts show a higher prevalence of approximately 13.3 to 15 per 100,000 [2]. This disparity is most pronounced in indigenous populations, where the prevalence fluctuates between 4.8 and 18.3 per 100,000 cases in New Zealand, Alaska, Australia, and Canada [2].

The growing recognition of the global burden of CSLDs in children has exposed significant inequities in resources and a lack of standardized management across health settings. To address these deficiencies, a recent international consensus established a rigorous set of quality standards to enhance pediatric patients’ quality of life. Individualized airway clearance techniques (ACTs) provided by a respiratory physiotherapist specialist are integral to these quality statements, as ACTs are considered a cornerstone of comprehensive CSLD management [3]. Moreover, the international roadmap of clinical priorities has revealed that access to physiotherapy and expertise in home-based equipment use are among the highest-rated needs expressed by children and their parents [4].

However, traditional delivery models often face substantial barriers, such as geographical distance and high treatment burdens, which can lead to suboptimal adherence and limited access to specialized expertise. While experts have suggested that digital health services may facilitate accessibility, there remains a critical requirement for evidence-based strategies to substantiate and refine the clinical utility of remote interventions within this population [5].

Digital health services (DHSs) cover a broad range of technologies for healthcare delivery, including telehealth (remote care), mHealth (mobile apps), remote patient monitoring (device data tracking), and digital therapeutics (software-driven interventions), aiming to enhance clinical outcomes [6]. In detail, they target compliance with the ACTs [7], the supervision of tailored exercise regimens [8,9,10,11,12,13], and the dissemination of structured patient education [14].

Building on the diverse utility of DHSs, a recent systematic review has rigorously evaluated the efficacy of delivering structured exercise programs via DHSs in children and adolescents with CSLDs [15]. Diverse interfaces, including active video games (*n* = 3), videoconferencing (*n* = 3), and digital spirometry (*n* = 1), were used to deliver and monitor exercise regimens in either in-person, remotely supervised at the clinic, or at-home settings in a pediatric population aged between 9 and 20 years old. A meta-analysis revealed a statistically significant improvement in the 6-minute walk test distance, with a pooled mean difference of 37.2 m, in favor of the eHealth group compared to the control group, who received the usual care [15]. However, studies included in this systematic review mainly referred to the pediatric population with cystic fibrosis (5 out of 7), highlighting the scarcity of the literature in other CSLD populations, such as PCD, PBB, or NCFB.

Adherence to ACTs remains a key component of disease management. Despite its clinical significance, the evaluation of adherence is limited, with the existing literature predominantly focused on pediatric cohorts with cystic fibrosis (CF). Available data indicate that adherence rates for unsupervised, home-based ACTs are consistently low [16]. Geographical distance from specialized physiotherapy centers constitutes a significant determinant of healthcare utilization and accessibility in physiotherapy [16]. Despite the increasing use of DHSs, there are no randomized controlled trials (RCTs) specifically targeting ACT adherence and access to care in CSLD populations other than those with CF.

This study aims to investigate the effects of applying digital physiotherapy services in the management of specific clinical outcomes in children with chronic suppurative lung diseases other than cystic fibrosis. We hypothesize that a hybrid (synchronous and asynchronous) tele-rehabilitation program that includes exercise training and ACTs can improve functional and exercise capacity, as well as compliance with ACTs, compared to the usual care. From the range of available DHSs, this study employed live videoconferencing sessions and an informative website.

## 2. Materials and Methods

### 2.1. Study Design

**Figure 1:**
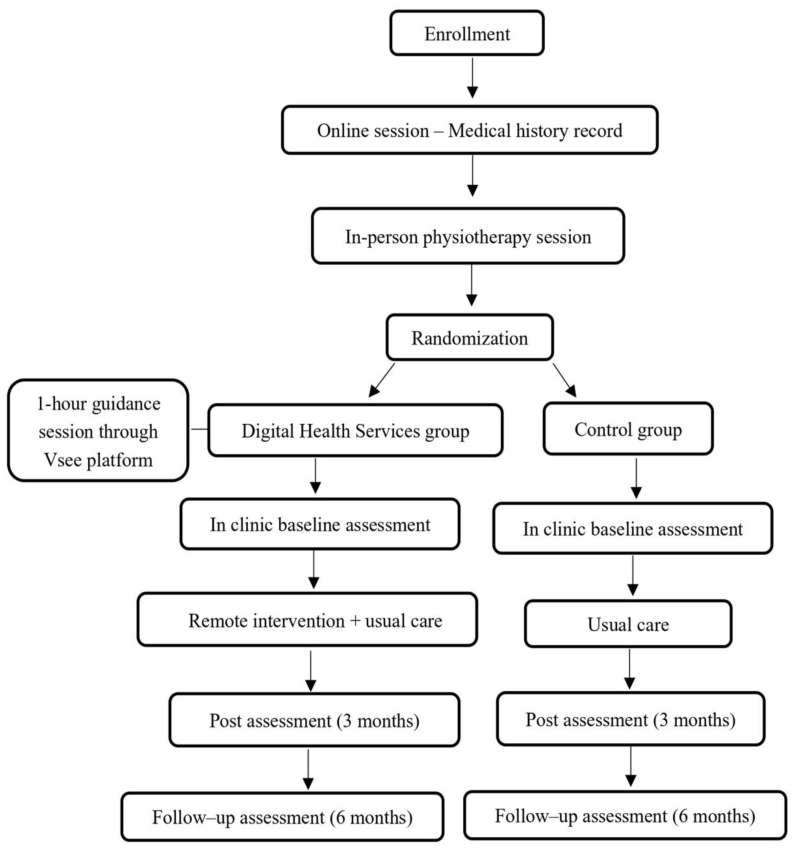
Study flow chart.

This is a prospective, single-center, two-arm (1:1), assessor-blinded, randomized controlled clinical trial that aims to recruit 32 children from the outpatient clinic of the third Pediatric Pulmonology Unit, “Attikon” University Hospital of Athens in Greece, between May 2026 and May 2027. To account for the geographical dispersion of patients across remote regions of Greece, the collection of medical histories will be conducted via a 40 min synchronous videoconference session through Microsoft Teams. Afterwards, all child–parent/caregiver dyads will attend a 40 min, in-person session with the physiotherapist at the outpatient clinic. This session is designed to provide standardized training on ACTs for subsequent implementation at home, as part of the usual care. Then, children will be randomized into parallel arms: the digital health services group (DHSG) and the control group (CG). All clinical assessments (baseline, post-intervention, follow-up) will be conducted in-person at the outpatient clinic by blinded healthcare professionals. All children will receive the usual care, including inhaled antibiotics and/or bronchodilators for respiratory infections, alongside recommendations to maintain regular physical activity and to perform ACTs at home. The DHSG will undergo a 12-week home-based hybrid (synchronous and asynchronous), remotely administered exercise program and ACTs. Likewise, the DHSG will have access to a specifically designed website about disease management (www.fysao.gr) [17]. Prior to the intervention, parents/caregivers in the DHSG will participate in a 1 h guidance session to ensure proficiency with the use of the VSee Clinic web platform (Vsee Lab, Inc., San Jose, CA, USA). VSee was selected for this study due to its secure, high-resolution videoconferencing software, ease of use, and the fact that it does not need a software system installation. The study process is presented in Figure 1. This protocol follows the Standard Protocol Items: Recommendations for Interventional Trials (SPIRIT) 2025 (Table 1) [18], and ethical approval has been provided by the Physiotherapy department of the University of Thessaly (ID-22510/2025) and the University Hospital of Athens “Attikon” (ID-517/2025) Clinical Research Ethics Committees. The protocol has been registered at ClinicalTrials.gov under identifier NCT07376187, submitted on 28/1/2026.

### 2.2. Randomization

To ensure blinding, an external individual who is not involved in the study will allocate participants randomly to each group using the randomizeR—R package, Version 1.4.2 (RWTH Aachen University, Aachen, Germany) (1:1 simple randomization), executed on an Apple macOS system [19], and will prepare the concealed opaque envelopes that will be given to the participants. Due to the nature of the study, the participants cannot be blinded. However, the study physiotherapist who will assess outcome measures, as well as the researcher who will perform the data analysis, will be blinded to participants’ group allocation.

### 2.3. Study Population

The study population consists of children aged 6 to 12 years old with CSLDs other than CF. Specifically, the inclusion criteria will target the diagnostic subgroups of PCD, PBB, and NCFB. Written informed consent will be obtained from all the children and their parents or legal guardians.

### 2.4. Eligibility Criteria

Eligible children will be screened based on a diagnosis of PCD, PBB, and NCFB, established via medical history, clinical features, or/and computed tomography and bronchoscopy findings. Inclusion criteria require children to be aged 6 to 12 years old, clinically stable with an absence of pulmonary exacerbation four weeks before the study’s recruitment, and maintain adherence to attending regular medical follow-up from a pediatric pulmonologist every three months. Regarding the diagnostic scope, children with cystic fibrosis will be excluded. Furthermore, children with immunodeficiency or asthma as their primary condition will also not be included. Additional exclusion criteria include children with clinical evidence of cardiovascular, neuromuscular, metastatic, or psychiatric comorbidities, and neuromuscular or musculoskeletal impairments that affect mobility and the ability to follow instructions. Ineligible children will also include those who have a medical history of lung transplants, who participate in other rehabilitation programs or attend regular physiotherapy ACT sessions (>10 sessions in a 3-month period), lack access to the internet (e.g., no smartphone, tablet, or laptop), or children and/or caregivers who are unable to use technological devices.

### 2.5. Procedure

#### 2.5.1. Intervention

##### Digital Health Services Group (DHSG)

Children randomized to the DHSG will engage in a home-based hybrid (synchronous and asynchronous), remotely administered exercise program and ACTs for 12 weeks. The prescribed weekly regimen will consist of 50 min remote sessions: two synchronous, supervised sessions conducted via the Vsee platform, and a minimum of one asynchronous, unsupervised session. Children will be encouraged to maintain daily adherence to the exercise protocol.

Secure access to the VSee platform will be granted through a unique identification code assigned to each child. Parents/guardians will receive written and verbal instructions regarding the Vsee platform setup, the equipment’s proper use, and safety procedures, including “actions in an emergency” in case of adverse events or technical issues. Adverse events are defined as unintended injuries or clinical incidents resulting from study participation. These include complications such as paroxysmal cough, severe dyspnea, oxygen desaturation (4% drop in SpO_2_), musculoskeletal injury, or loss of balance. To ensure safety in any adverse event (e.g., falls, injury, etc.), at least one parent or guardian will be required to be present in person with the child during sessions. Additionally, written first-aid advice will be given. All adverse events will be recorded during the synchronous videoconferencing sessions. The physiotherapist will document the nature, severity, and duration of each event in a standardized log.

Children will perform ACTs (including individualized teaching and review of postural drainage, percussion, vibration, ACT devices, huffing, coughing), diaphragmatic breathing, and blowing games to enhance mucus expectoration. Both children and parents will be taught how to perform these techniques. During the synchronous, supervised videoconference sessions, the DHSG will be monitored/supervised by the pediatric respiratory physiotherapist for the proper performance of ACTs (device or non-device). All children will perform ACTs once a day for ten minutes. In case of mucus excess, they will be suggested to increase the performance to twice a day. ACTs will be chosen based on children’s ability to use/cooperate and preferences.

Building upon a previous study in children with CF [11], our research team designed an exercise program based on the 24-letter Greek alphabet, where each letter represents an individual aerobic or strengthening exercise, selected so that they can be implemented easily in an online setting at home with minimal equipment. For example, the Greek letter “A” represents the exercise “ALMA”, meaning jumping up and down. In order to adapt the exercise program to age-specific needs and capabilities, the exercises have been modified for two age groups: 6–9 and 10–12 years old. The exercise program consists of 16 exercises per session, varied on the four-letter words chosen at a time. At each session, a combination of four words will be performed and modified weekly. All children will follow the same word combination every week. The exercise program will last 25 to 30 min per session.

The exercise workload will be equal to 60–75% of the maximum heart rate (HR) [20]. The target heart rate (THR) will be calculated based on the following Karvonen formula [21]:

THR = [(HR_max_ − HR_rest_) × % intensity] + HR_rest_

Exercise parameters (heart rate, oxygen saturation) will be measured before and at the end of the exercise program by the parents/guardians attending the session. Dyspnea and leg fatigue will be evaluated using the Dalhousie Pictorial Scale [22]. The exercise program will be delivered and monitored by a pediatric respiratory physiotherapist.

Additionally, children and their parents/caregivers will be granted access to a website (www.fysao.gr) for disease management, including information about common symptoms, nutrition, pharmacological treatment, exercise, and ACT performance. The DHSG will utilize this specifically designed digital interface, the development and structural framework of which have been previously detailed by our research team [17]. To facilitate adherence and longitudinal monitoring, the DHSG will receive automated weekly reminders for ACTs and exercise performance. Through the web portal’s integrated evaluation forms, children can report daily symptomatology and track any clinical fluctuations. All the ACTs and the exercises have been digitized as high-definition videos and images, ensuring asynchronous accessibility for the DHSG. Furthermore, the platform facilitates secure communication, enabling children and their parents/caregivers to communicate with healthcare professionals via integrated email or direct document transmission.

##### Control Group (CG)

Participants in the control group will receive the usual care, typically comprising inhaled antibiotics and/or bronchodilators for respiratory infections as regular pharmacological treatment in clinically stable condition and a 40 min, in-person, physiotherapy session where they will be taught ACTs to perform at home. A printed copy of a handbook, including information on their disease and symptoms, nutrition, pharmacological treatment, exercise, and how to perform ACTs, will be provided.

### 2.6. Remotely Administered Educational Session

The DHSG will receive a remote synchronous session of 1 h with guidance regarding familiarization with the use of the VSee platform. The Vsee platform is a secure, high-resolution videoconferencing software. It is easy to use, and it does not need to download any software system to the device. During this session, a verbal presentation will be carried out on how children and parents have to use the equipment to measure their heart rate, oxygen saturation, and how they should assess dyspnea and leg fatigue with the Dalhousie Pictorial Scale. Furthermore, parents/guardians will be trained on the proper use of the above equipment. Additionally, verbal instructions will be given to minimize difficulties relating to internet connection.

### 2.7. Assessment Procedure

All outcome measures will be assessed at the following time points: baseline (before the intervention), the end of the intervention (12 weeks), and follow-up (6 months after the intervention has ended). Assessment procedures will be performed in person at the hospital by a blinded, independent assessor (physiotherapist) on two consecutive days to avoid participant fatigue. Anthropometric characteristics, including weight, height (to calculate body mass index, BMI), fat mass (kg and %), and fat-free mass, will be measured using a stadiometer and the digital fat mass scale TANITA RD-545 (Tanita Corporation, Tokyo, Japan). Pulmonary function will be assessed through the performance of spirometry (Jaeger Vyntus Pneumo, Vyaire Medical, Mettawa, IL, USA) and the forced oscillation technique (FOT) (Jaeger Vyntus IOS, Vyaire Medical, Mettawa, IL, USA) [23,24]. Functional and exercise capacity will be assessed using the 6-minute walking test, the modified shuttle walk test, and the Chester step test. Respiratory muscle strength, handgrip, health-related quality of life (HRQoL), cough-specific QoL, physical activity (PA), and sedentary behavior will be assessed. Furthermore, twice a month for both groups, ACT performance and symptom behavior will be recorded in a detailed diary.

### 2.8. Outcome Measurement

#### 2.8.1. Primary Outcome

##### Exercise Capacity (Modified Shuttle Walk Test)

Maximal exercise capacity will be assessed through the modified shuttle walk test (MSWT). Its reliability has been previously evaluated in the pediatric population with CF [9]. In pediatric populations with CF, a 60 m improvement in the MSWT following a 6-week home-based exercise program has been identified as the minimal clinically important difference [25]. During the MSWT, participants will be asked to walk rapidly at gradually increasing speeds (15 levels total) along a 10 m corridor [26,27]. An audio signal (“beep”) will mark the transitions between levels, signaling a required increase in velocity. The protocol will commence at a baseline speed of 0.5 m/s (Level 1), with an incremental increase of 0.17 m/s for each subsequent level. The test will be terminated based on inability to continue, symptomatic fatigue, or the failure to reach the course marker before the auditory signal on two consecutive occasions. The walking distance (MSWD) will be recorded, and performance will be compared based on the equation proposed by Pinho et al. [28]. Two trials will be performed, with at least a 30 min rest. Before, during, and after (recovery period), cardiorespiratory parameters will be continuously recorded.

##### Compliance with the Airway Clearance Techniques and Exercise Program

Compliance with the ACTs will be recorded using a detailed diary that includes weekly symptoms, type, frequency, and duration of ACTs performed. For both groups, compliance will be assessed twice a month, following the completion of this diary. The DHSG will complete the diary through the website, while the CG will complete it in a paper version via a phone call from a physiotherapist. Furthermore, the DHSG’s exercise program compliance will be assessed every second online session through a Microsoft Form diary. Documentation of the percentage of completed synchronous videoconferencing sessions will also be performed.

#### 2.8.2. Secondary Outcomes

##### Functional and Exercise Capacity

The 6-minute walking test (6MWT) will be used to assess functional capacity. The participants will be asked to walk as far as possible in a 30 m corridor, and standardized encouragement will be given after each minute [29]. The total walking distance (6MWD) will be recorded, and predicted values will be used based on Ulrich et al.’s study [30].

Apart from the MSWT, maximal exercise capacity will be assessed with the Chester step test (CST). The CST is a multi-staged test that requires participants to step on and off a 20 or 25 cm tall step with no handles at a rate set by a metronome beat, which is continuously increasing [31]. Due to the range of ages (6 to 12 years old), these step heights were chosen to be optimal for children according to their age [32]. It consists of 5 levels, each of a two-minute duration. The total number of steps and the maximal level achieved will be recorded. Before, during, and after (recovery period) all tests, cardiorespiratory parameters will be recorded.

Both the 6MWT and CST will be performed twice, with at least 30 min rest. To avoid fatigue, 6MWT will be performed prior to the maximal exercise tests.

##### Health-Related Quality of Life (HRQoL)

Pediatric Quality of Life Inventory Version 4.0 (PedsQL) is a self-administered questionnaire that includes 23 items divided into four subscales: Physical Functioning (PH), Emotional Functioning (EM), Social Functioning (SOC), and School Functioning (SCH) [33]. Responses are given on a five-point scale ranging from 0 (never a problem) to 4 (almost always a problem) and are reverse-scored so that lower scores reflect more negative functioning.

#### 2.8.3. Exploratory Outcomes

##### Respiratory and Peripheral Muscle Strength

Maximal inspiratory (MIP) and expiratory (MEP) pressure will be measured using a mouth pressure meter (MicroRPM; MicroMedical, Lewiston, ME, USA) to assess respiratory muscle strength [34]. A maximum value of three efforts with a variation of less than 5% will be recorded for both inspiratory and expiratory pressures. There will be a minute of rest between the efforts.

Handgrip strength will be assessed using a hand dynamometer (JAMAR, Patterson Medical, Warrenville, IL, USA). Participants will hold the dynamometer at a 90° angle to their elbow. Three separate efforts for the dominant hand grip (DHG) and three for the non-dominant hand grip (NDHG) will be administered, with 30 s of rest between efforts. The highest value in kilograms (kg) for each hand will be recorded [35,36].

##### Physical Activity and Sedentary Behavior

The Physical Activity Questionnaire for Older Children (PAQ-C) is a ten-item self-administered questionnaire that intends to measure PA [37]. Nine of the ten items are scored on a five-point rating scale, where higher scores indicate a higher level of activity. The first item of the PAQ-C consists of 22 common sports and leisure activities for which the participants select the score based on the frequency of the activities performed during the preceding seven days on a five-point rating scale (1 = no activity at all, 2 = 1–2 times, 3 = 3–4 times, 4 = 5–6 times, and 5 = 7 times or more) after a mean composite score is calculated. The remaining eight items address PA performed during the day (e.g., physical education classes, recess time, lunchtime, and after-school activities on weekday evenings and weekends) and a summary for all days of the week. The mean score of the first nine items is the summary score of the PAQ-C.

The Youth Activity Profile (YAP) comprises 15 items in total, each scored on a 5-point scale designed to capture four components in youth behaviors: (1) PA in school, (2) PA out of school, (3) PA over the weekend, and (4) sedentary behaviors [38]. Each YAP section is developed to be scored independently (i.e., items for each dimension score would reflect a higher expected activity level/sedentary time in that same dimension).

##### Cough-Specific Quality of Life (CC-QoL)

The Cough-specific Quality of Life (CC-QoL) is a 7-day recall questionnaire designed to reflect several domains related to a child’s cough, including physical (e.g., coughing makes you feel tired), social (e.g., others staring at you), and psychological (e.g., feeling upset) factors [39]. The responses are rated on a 7-point Likert scale (1 = all the time to 7 = none of the time), with higher scores reflecting higher QoL.

##### Digital Health Services Satisfaction

Satisfaction from the use of the VSee platform and FysAΩ website (www.fysao.gr) will be assessed using the Telemedicine Usability Questionnaire (TUQ). The TUQ consists of 21 questions, scored on a Likert scale from 1 (totally disagree) to 7 (totally agree) [40]. The total score is calculated by the mean of all 21 questions [41].

### 2.9. Sample Size Calculation

Due to the lack of pilot data for this specific population, the effect size was estimated a priori. We targeted a moderate effect size (f = 0.25), assuming that this represents a clinically significant improvement in exercise capacity for children with CSLDs. The required sample size was calculated with G*Power version 3.1.9.4 (G*Power, University of Düsseldorf, Düsseldorf, Germany). The “F-test” (ANOVA: repeated measures, within-between interaction) and the detection of a moderate effect size f = 0.25 (alpha level = 0.05, power = 80%) showed that a total of twenty-eight children are required. Assuming a dropout rate of 10%, the total sample size is estimated at thirty-two children, with sixteen in each group.

### 2.10. Statistical Analysis

Statistical analysis will be performed using IBM SPSS—Mac, Version 31.0 (IBM Corporation, Armonk, NY, USA). The normal distribution of the variables will be demonstrated using the “Shapiro–Wilk” test. The analysis will be performed using an intention-to-treat (ITT) approach, and all the randomized participants will be included (including those who may discontinue the study or be noncompliant with the assigned therapy). Reasons for discontinuing the study will be recorded. Missing data will be handled through a two-stage approach. Initially, we will re-contact (recall) all participants who have incomplete data. For the remaining missing values, multiple imputation (MI) will be employed [42]. Parametric and non-parametric analyses will be performed, based on the normal distribution of the variables. A separate 2 × 3 mixed-model analysis of variance (ANOVA) will be used to examine the effects of the intervention on the primary and secondary outcomes. For the group × time interaction in the ANOVAs, the factors that will be analyzed are group (DHSG and CG) and time (baseline, end of treatment, and follow-up). For this test, a partial eta-squared (η^2^) of 0.01–0.059 represents a small effect, a value of 0.06–0.139 represents a medium effect, and a value >0.14 represents a large effect. Differences between measurements will be analyzed using Bonferroni correction. A *p*-value < 0.05 will be considered statistically significant for all the analyses.

## 3. Discussion

This protocol outlines a 12-week intervention that combines ACTs and exercise training delivered through synchronous (videoconferencing) and asynchronous implementation at home, supported by an informative, specially designed website and supervised by a pediatric respiratory physiotherapist in non-CF CSLD populations.

Effective ACTs are consistently identified by both clinicians and caregivers as a fundamental component of managing CSLDs [4]. This protocol explores a hybrid digital approach to the physiotherapy management of non-CF CSLDs by integrating DHSs into everyday physiotherapy care and aiming to enhance adherence, which is often suboptimal in children and adolescents with CSLDs [43].

DHSs in pediatric respiratory care are an expanding field, aiming to provide tailored support to the special needs of this population [44]. While digital interventions have shown potential in improving pulmonary function, exercise capacity, balance, periphery, and respiratory muscle strength and HRQoL in children and adolescents with CF, with a meta-analysis reporting a mean improvement of 37.2 m in the 6MWT [15], there is limited evidence in non-CF CSLD subgroups.

The primary hypothesis is that this hybrid tele-rehabilitation program will improve exercise capacity and compliance with ACTs compared to the usual care. This hypothesis is based on the integration of synchronous supervision, which aims to maintain appropriate exercise intensity, a factor that may be compromised in conventional, unsupervised home-based care. Furthermore, the “FysAΩ” website is designed to support adherence to ACTs by providing digitized instructional resources regarding exercise and ACT performance, potentially mitigating the high non-adherence rate typically observed in pediatric CSLDs [4].

A key aspect of this study lies in its novelty, focusing on non-CF CSLD populations and expanding the existing evidence base through a rigorous methodological framework. Due to the targeted sample size, secondary outcomes beyond functional and exercise capacity (CST), as well as HRQoL, are classified as exploratory. This approach prioritizes the study’s primary objectives while exploring the preliminary impact of this hybrid tele-rehabilitation on this population. Additionally, the feasibility of the protocol is supported by the research team’s previous clinical experience in this field, participating in home-based hybrid (synchronous and asynchronous), remotely administered assessment and rehabilitation protocols in chronic diseases, using technologies such as the Vsee platform [45,46,47]. Despite this protocol referring to a single-center design, this hybrid digital structure that combines synchronous videoconferencing with an asynchronous website is designed to allow for real-time corrective feedback for ACTs while addressing geographical barriers to specialized care. Ultimately, the results of this study may contribute new insights into the integration of digital health modalities in the management of this pediatric population by physiotherapists.

## Figures and Tables

**Table 1:** Standard protocol items: recommendations for interventional trials (SPIRIT) 2025.

	Enrollment	Allocation	Baseline	12 Weeks	6 Months
**Enrollment**					
Eligibility screen	X				
Informed consent	X				
Online medical history	X				
Physiotherapy session	X				
Randomization		X			
**Intervention/Comparator**					
Digital health services group (DHSG)			X	X	
Control group (CG)			X		
**Assessments**					
**Characteristics**					
Anthropometrics (BMI, fat mass)			X	X	X
Pulmonary function (spirometry/FOT)			X	X	X
**Primary outcomes**					
Exercise capacity (MSWT)			X	X	X
Compliance (ACTs and exercise)			X	X	X
**Secondary outcomes**					
Functional capacity (6MWT)			X	X	X
Exercise capacity (CST)			X	X	X
Health-related quality of life (PedsQL)			X	X	X
**Exploratory outcomes**					
Respiratory muscle strength (MIP/MEP)			X	X	X
Peripheral muscle strength (Handgrip)			X	X	X
Physical activity (PAQ-C, YAP)			X	X	X
Cough-specific quality of life (CC-QoL)			X	X	X
Digital health services satisfaction (TUQ)				X	

6MWT: 6-minute walking test; ACTs: airway clearance techniques; BMI: body mass index; CC-QoL: cough-specific quality of life; CG: control group; CST: Chester step test; DHSG: digital health services group; FOT: forced oscillation technique; MEP: maximal expiratory pressure; MIP: maximal inspiratory pressure; MSWT: modified shuttle walk test; PAQ-C: physical activity questionnaire for older children; PedsQL: pediatric quality of life inventory version 4.0; TUQ: telemedicine usability questionnaire; X: assessment timepoint; YAP: youth activity profile.

## Data Availability

No new data were created or analyzed in this study.

## Abbreviations

The following abbreviations are used in this manuscript:

6MWD	6-minute Walking Distance
6MWT	6-minute Walking Test
ACTs	Airway Clearance Techniques
BMI	Body Mass Index
CSLDs	Chronic Suppurative Lung Diseases
CG	Control Group
CF	Cystic Fibrosis
CST	Chester Step Test
CC-QoL	Cough-Specific Quality of Life
DHG	Dominant Hand Grip
DHSG	Digital Health Services Group
DHSs	Digital Health Services
EM	Emotional Functioning
FOT	Forced Oscillation Technique
HR	Heart Rate
HRQoL	Health-Related Quality of Life
ITT	Intention-to-treat approach
MEP	Maximal Expiratory Pressure
MI	Multiple Imputation
MIP	Maximal Inspiratory Pressure
MSWD	Modified Shuttle Walk Distance
MSWT	Modified Shuttle Walk Test
NCFB	Non-Cystic Fibrosis Bronchiectasis
NDHG	Non-Dominant Hand Grip
PA	Physical Activity
PAQ-C	Physical Activity Questionnaire for Older Children
PBB	Protracted Bacterial Bronchitis
PCD	Primary Ciliary Dyskinesia
PedsQL	Pediatric Quality of Life Inventory Version 4.0
PH	Physical Functioning
QoL	Quality of Life
RCTs	Randomized Controlled Trials
SCH	School Functioning
SOC	Social Functioning
SPIRIT	Standard Protocol Items: Recommendations for Interventional Trials
THR	Target Heart Rate
TUQ	Telemedicine Usability Questionnaire
YAP	Youth Activity Profile
